# Endoplasmic reticulum stress induced autophagy alters cellular processing of cationic lipid delivered siRNAs

**DOI:** 10.1007/s13346-026-02096-8

**Published:** 2026-03-17

**Authors:** R. Chauncey Splichal, Christina Chan, S. Patrick Walton

**Affiliations:** 1 Department of Chemical Engineering and Materials Science, Michigan State University, East Lansing, MI USA; 2 Department of Biochemistry and Molecular Biology, Michigan State University, East Lansing, MI USA; 3 Department of Computer Science and Engineering, Michigan State University, East Lansing, MI USA; 4 Institute of Quantitative Health Science and Engineering, Michigan State University, East Lansing, MI USA

**Keywords:** SiRNA delivery, Autophagy, ER stress, Disease State

## Abstract

siRNA therapeutics have achieved increased clinical success in the past decade. To achieve a therapeutic effect, siRNAs must enter the cytoplasm of the target cells. Thus, their function typically relies on proper endocytosis and trafficking. Endoplasmic reticulum (ER) stress is associated with diseases that are being studied for treatment with siRNAs (cancer, amyloids, mutations, etc.) and common comorbidities (obesity, smoking, hypertension, etc.). ER stress results in activation of the Unfolded Protein Response (UPR), which initiates changes in cellular function that include disruption to endosomal vesicle trafficking and processing. We used the N-glycosylation inhibitor, tunicamycin, to induce ER stress in HeLa cells during transfection with siRNAs targeting EGFP. Our results showed that ER stress resulted in an increased accumulation of siRNA but a reduction in silencing of the siRNA target (EGFP). Further, we used 3-methyladenine (autophagy inhibitor) and bafilomycin A1 (inhibits endosome maturation) to show that autophagy plays a role in increasing siRNA accumulation. Finally, we showed that the additional accumulated siRNA in ER-stressed cells remained functional, was retained in cells for longer, and prolonged silencing. Our results will inform the design of siRNA delivery vehicles and dosing schedules, by ensuring that disease complications, specifically ER stress/UPR activation, are considered.

**Graphical abstract:**

**Figure:**
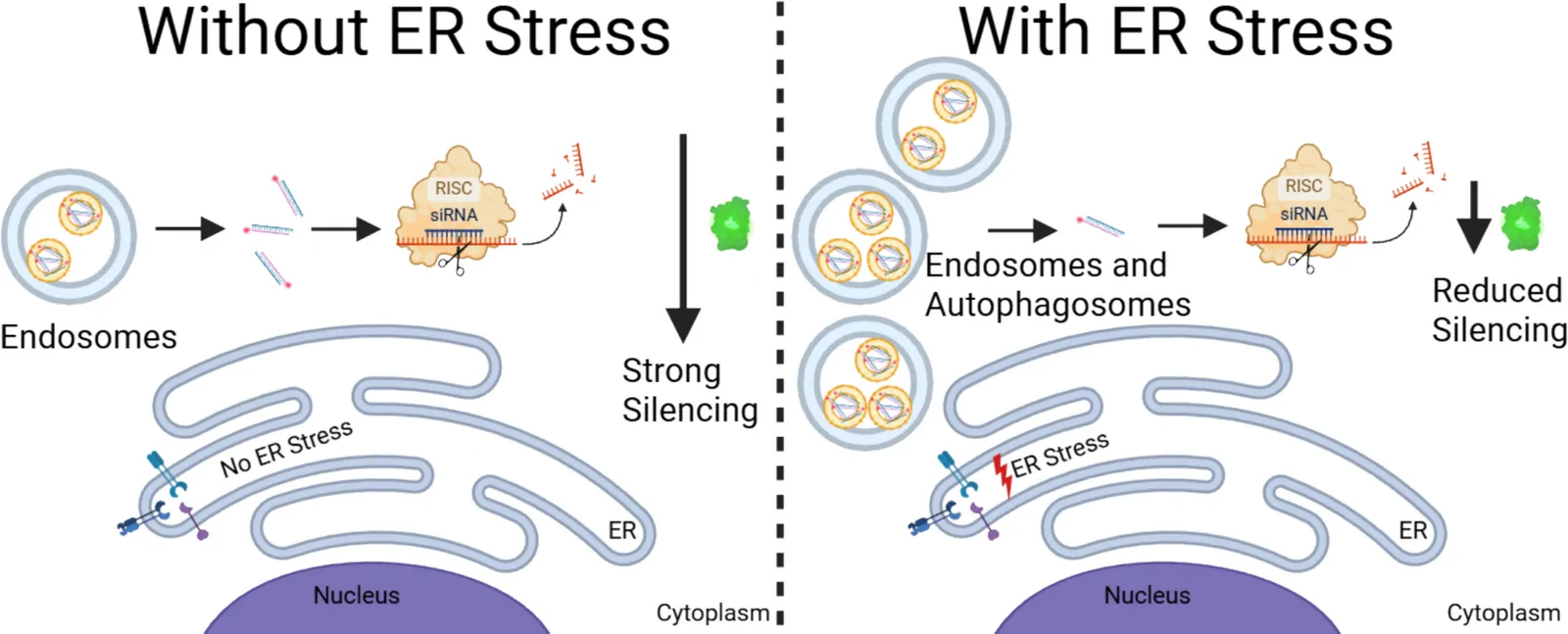

**Supplementary Information:**

The online version contains supplementary material available at https://doi.org/10.1007/s13346-026-02096-8.

## Introduction

Small interfering RNAs (siRNAs) are an increasingly important class of therapeutics, with seven FDA-approved therapeutics and 156 active or recruiting clinical trials [1–8]. siRNAs are large (compared to small molecule drugs), charged molecules and must be modified or complexed with a delivery vehicle to enter the cytoplasm of a target cell and achieve a therapeutic effect [9]. Disruptions to endocytosis and intracellular processing from common comorbidities such as obesity, diabetes, smoking, and hypertension could therefore affect siRNA activity [10], principally through disruption of the function of the endoplasmic reticulum (ER). The ER is a primary controller of membrane turnover and endocytosis, which are required for uptake and trafficking of siRNAs. In response to stress (such as the comorbidities mentioned above), cells upregulate synthesis of defensive proteins, including chaperones, cytokines, and metabolic enzymes [11]. In many cases, the increased protein synthesis burden overwhelms the quality control systems of the ER, resulting in an accumulation of unfolded or misfolded proteins, a condition broadly labeled ER stress [12, 13].

In response to ER stress, the unfolded protein response (UPR) is initiated by three sensor proteins: inositol-requiring enzyme 1 α (IRE1α), protein kinase R (PKR)-like ER kinase (PERK), and activating transcription factor 6 (ATF6). To restore protein homeostasis, the UPR downregulates protein translation, increases chaperone protein expression to refold misfolded proteins, and increases protein degradation of unfolded and misfolded proteins that cannot be rescued. If these changes fail to restore proteostasis, apoptosis is initiated [11]. In the presence of ER stress, IRE1α dimerizes and trans-autophosphorylates, activating an RNase domain that splices XBP1 mRNA leading to expression of an active transcription factor (XBP1s) [14]. XBP1s activates the JNK pathway to induce apoptosis and ER associated degradation (ERAD) of proteins and autophagy [15]. PERK is activated upon dimerization after immunoglobulin-binding protein (BiP) dissociation. Activated PERK subsequently phosphorylates eIF2α, which inhibits mRNA translation, and activates ATF4. ATF4 increases expression of target genes regulating autophagy including autophagy regulating gene 5 (ATG5), ATG12, and ATG16L [16]. Upon dissociation from BiP, ATF6 translocates to the Golgi, where proteases site-1-protease (S1P) and site-2-protease (S2P) cleave ATF6 into the active transcription factor ATF6f, which upregulates XBP1 and C/EBP Homologous Protein (CHOP) further increasing autophagy [17]. ER stress is induced by many different effectors; thus, ER stress does not always coincide with an activation of autophagy. Current knowledge is that ER stress will have an increasing effect on autophagy unless the stress is too severe or too prolonged, in which case apoptosis is initiated and autophagy is inhibited [17–19].

To achieve silencing, siRNAs must enter the cytoplasm, typically by crossing the endosomal membrane after endocytosis [20, 21]. The exact mechanism of endosomal escape of cationic lipid-delivered siRNAs is unclear, but there is evidence supporting the role of pore formation or membrane fusion in endosomal escape, as well as direct siRNA transport into the cytoplasm [21–23]. Regardless, delivery to the cytoplasm involves electrostatic interactions between the siRNA-containing complexes and a membrane [21]. The lipid content of membranous vesicles is determined by lipid synthesis that occurs in the ER and also changes due to ER stress [24]. Sterol Regulatory Element Binding Protein 1 (SREBP1) and SREBP2 translocate along with ATF6 to the Golgi, where they are cleaved by S1P and S2P to become active transcription factors that increase cholesterol synthesis, thereby increasing the rigidity of cellular membranes as a pro-survival means to impede transport of stress-causing material across membranes; thus, the transport of other materials, including siRNAs, is also impeded [25]. IRE1α increases Diacylglycerol O-acyltransferase 2 (DGAT2), Stearoyl-CoA Desaturase (SCD1), and Acetyl-CoA Carboxylase (ACC2) expression, which are involved in the synthesis of triglycerides, monounsaturated fatty acids, and saturated fatty acids, respectively [26, 27]. As with cholesterol synthesis, changes in fatty acid composition and content also alter membrane rigidity [28, 29]. PERK’s activation of ATF4 leads to increased expression of SREBP1 and SCD1 [30]. In addition to the UPR altering membrane rigidity, increases in membrane rigidity can induce the UPR because increased lipid packing increases IRE1α and PERK dimerization and activation [31]. Overall, cells experiencing ER stress tend to have more rigid, less fluid membranes [31].

In addition to altering membrane content and biophysical properties, ER stress can alter the trafficking of endomembrane vesicles by increasing autophagy. Autophagy is a protective mechanism initiated by all three branches of the UPR to degrade unfolded proteins, increase energy production, and sequester stress-causing material [19]. There are numerous reported mechanisms for autophagy initiation [32]. After an initiation event, membrane lipids and proteins from the ER are recruited to form a phagophore that elongates to surround the material to be degraded. When the phagophore has fully engulfed the material, it becomes a double-bilayer autophagosome, which eventually merges with a lysosome to degrade the encapsulated contents [33]. Autophagy impacts endocytosis through depletion of shared membrane resources [34]. Phagophore elongation requires lipids from nearby membranes including endosomes and the ER [34]. Autophagosomes can also merge with Rab-coated (endosome trafficking) and COPII-coated (ER-Golgi transport) vesicles. Under ER stress, autophagy-related (ATG) proteins can be transported to existing vesicles to convert them into autophagosomes [35]. Generally, autophagy removes lipids, proteins, and vesicles from the rest of the endomembrane system, potentially altering siRNA endocytosis, trafficking, and function.

Given the multifaceted effects of ER stress on the endomembrane system, in this study, we wanted to investigate the impact of tunicamycin (TM)-induced ER stress on the accumulation and effectiveness of siRNAs delivered to HeLa cells. ER stress increased siRNA accumulation but, at short times, reduced silencing. We identified autophagy as a major contributor to the increase in siRNA accumulation. Finally, we demonstrated that the accumulated siRNA in ER-stressed cells remain functional and extends the duration of silencing.

## Methods

### Cell culture

HeLa-EGFP cells were provided by Dr. Manfred Gossen^32^. Cells were passaged via trypsinization three times per week and maintained in antibiotic-free Dulbecco's Modified Eagle Medium (DMEM, Gibco 119,625), 10 mM HEPES, and 10% FBS at 37 °C and 5% CO_2_.

### EGFP silencing

HeLa-EGFP cells were plated in 24-well plates (Corning 3526) at 90,000 cells/mL and 0.5 mL per well. 24 h after plating, culture media was replaced with serum-free DMEM containing tunicamycin (TM) (MP Biomedicals, 11,089–65-9), bafilomycin A1 (BAFA1) (MSPP-TLRLBAF1, VWR), 3-methyl adenine (3MA) (Sigma, SAE0107) or a combination. For one hour, cells were allowed to incubate in the serum-free media containing TM or BAFA1 while Cy5-siRNA/LF2K complexes were formed per manufacturer’s instruction. For the 3MA experiments, cells incubated in 5 mM 3MA containing serum-free media for 2 h before TM was added for an additional one hour treatment prior to transfection. Complexes were made to achieve a final concentration of 1.75 µg/mL LF2K (Invitrogen, 11,668,019) and 150 nM siRNA (Horizon Discovery, Guide strand sequence: GGCUACGUCCAGGAGCGCAUU) in 50 µL of Opti-MEM (Gibco, 31,985,062) and 500 µL of DMEM. Cells were incubated with the transfection solution for 4 h and then washed with 50 µg/mL heparan sulfate (AMSBIO, GAG-HS01) in 1 mL DPBS (Gibco, 14,190,144) for 10 min. Upon removal of the heparan sulfate, 0.5 mL of fresh culture media was added to the cells, and they were allowed to incubate for 24 h before being fixed for flow cytometry (see below).

To ensure that all the no siRNAs were adhered to the exterior of cells, immediately following transfection, cells were washed with polyanionic heparan sulfate. 24 h post heparan sulfate washing cells were fixed and taken to flow cytometry. In this paper, we define silencing to mean the percent reduction of EGFP signal in cells treated with siRNA compared to cells treated with all other media and transfection components with 0 nM siRNA (Eq. 1).

1 $$Percent \;Silencing=\left(1-\frac{EGFP \;RFU\; of\; cells \;treated \;with\; siRNA\&LF2K \;complexes}{EGFP \;RFU \;of \;cells \;treated \;with\; LF2K\; only}\right)*100\%$$

### Cell fixation and flow cytometry

Cells ready for flow cytometry were washed with 300 µL of Versene (Gibco, 15,040,066) before being removed from the plate with 200 µL of trypsin–EDTA (Gibco, 25,200,072). After 3 min of incubation with trypsin–EDTA at 37 °C, 2 mL of culture media were added to inactivate the trypsin. This solution was moved to a centrifuge tube and centrifuged at 220 rcf for 3.5 min. The supernatant was removed. Cells stained with Zombie Violet LIVE/DEAD (BioLegend) were stained for 15 min at room temperature in 100 µL at a ratio of 1:1000 Zombie Violet to DPBS. All cells were then washed with 5 mL of DPBS. Cells were then centrifuged for 3.5 min at 220 rcf before the DPBS was removed and replaced with DPBS containing 2% paraformaldehyde (Sigma, **158,127**) at 37 °C. Cells were then incubated in the fixative solution for 20 min before being centrifuged and rinsed twice with fresh DPBS. Finally, the cells were resuspended in 250 µL of sorting solution and run on an Attune CyPix flow cytometer. The flow cytometer measured of EGFP (ex. 489 nm/em. 511 nm) and Cy5 (ex. 651 nm/em. 670 nm; siRNA tag). Flow cytometry signal was gated using forward scattering area (FSC-A) and side scattering area (SSC-A) to identify round and appropriately sized material and eliminate debris. Next, cells were gated using SSC-A with side scattering width (SSC-W) to eliminate cell aggregates. The gain of EGFP and Cy5 channels on the flow cytometer was set so that no singlet cell was measured above 90% of the bit depth. siRNA accumulation was measured as the geometric mean of the raw fluorescence units (RFU) recorded by the cytometer. EGFP signal used in Eq. 1 was measured as the geometric mean of RFU recorded by the cytometer.

### Statistics

Unless otherwise stated, replicates were produced by plating cells into 3 different plates with 3 replicates on each plate. Each plate received individually prepared solutions of TM, siRNA/LF2K, and recovery media. Statistical analysis was first done by two-way ANOVA to determine whether any significant differences existed. This was followed by Student’s t-tests to calculate pairwise p-values and compared with Holm-Bonferroni corrected levels of significance for multiple comparisons.

## Results

### ER stress increased siRNA accumulation and decreased silencing

**Fig. 1:**
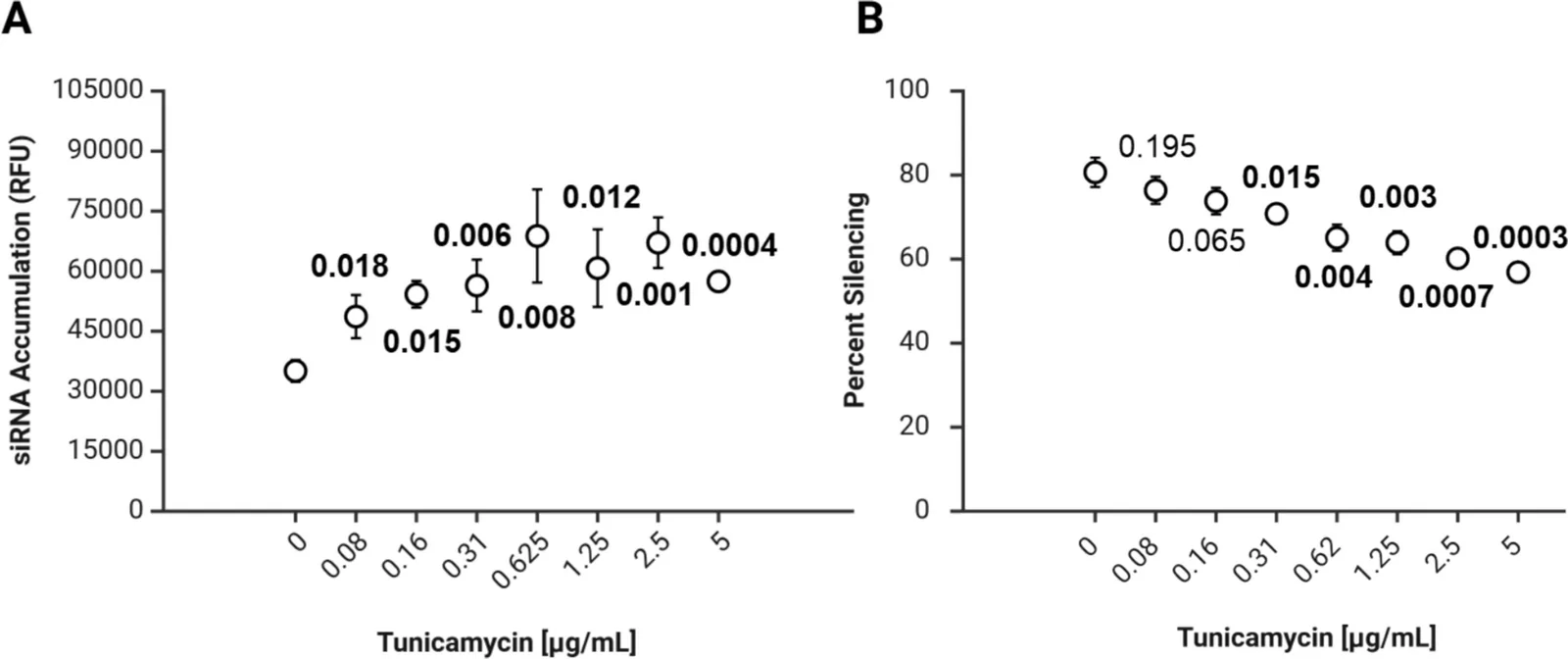
The impact of TM on siRNA accumulation and EGFP silencing. **A**) siRNA accumulated vs. TM concentration. **B**) EGFP silencing vs. TM concentration. Labels for each data point are the p-values of Student’s T-tests comparing the data point to the 0 µg/mL TM data point; bold indicates *p *< 0.05 and Holm-Bonferroni adjusted level of signifigance. Error bars represent standard deviation, N = 3

To determine the impact of ER stress on siRNA function, the N-glycosylation inhibitor tunicamycin (TM) was used to induce ER stress in HeLa cells that constitutively express EGFP. Transfection of a Cy5-tagged siRNA using Lipofectamine 2000 (LF2K) in cells treated with increasing levels of TM resulted in increasing accumulation of siRNA 24 h post transfection (Fig. 1A). Counterintuitively, increasing TM led to a decrease in silencing (Fig. 1B), despite the higher intracellular siRNA concentrations. This indicated that siRNAs entered the cell and were sequestered in a manner that prevented initiation of RNAi. Because TM is a potent inducer of ER stress through improper folding and glycosylation patterns, EGFP signal is impacted by TM; however, EGFP signal is reduced by siRNA silencing beyond reductions due to TM alone (Supplemental Fig. 1).

### Autophagy inhibition reduces ER stress-induced siRNA accumulation

**Fig. 2:**
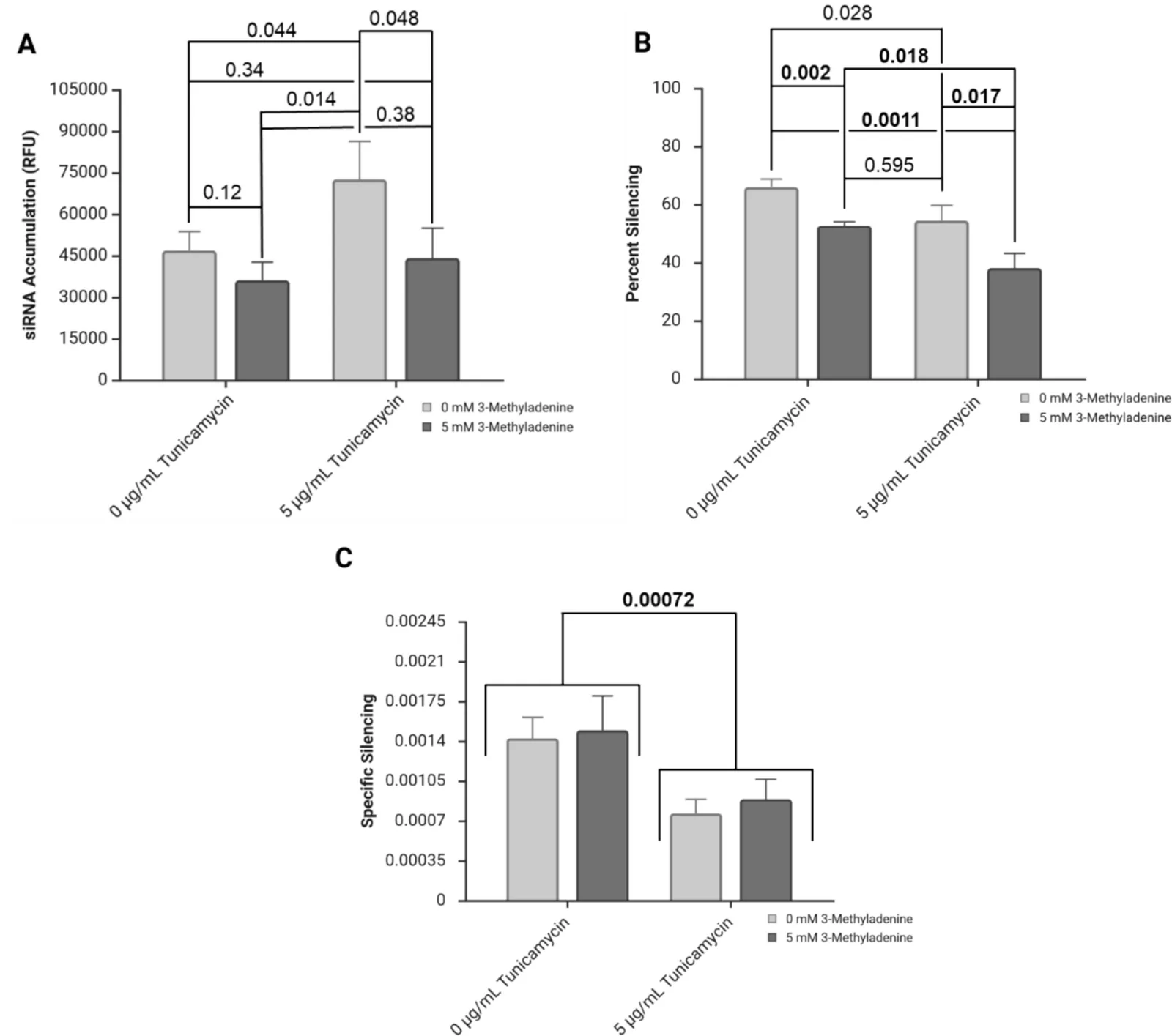
Effect of 3MA and TM on siRNA accumulation, silencing, and specific silencing. **A**) siRNA accumulation in control, 3MA treated, TM treated, and co-treated with TM and 3MA cells. **B**) Percent silencing in control, 3MA treated, TM treated, and co-treated with TM and 3MA cells. **C**) specific silencing (silencing divided by siRNA accumulated) in control, 3MA treated, TM treated, and co-treated with TM and 3MA cells. p-values are the result of Student’s T-tests comparing indicated values. **A**) All p-values are larger than Holm-Bonferroni adjusted level of significance. **B**) P-values shown in bold are less than Holm-Bonferroni adjusted level of significance. **C**) p-value resulting from two-way ANOVA, 3MA comparisons are not significant. Error bars represent standard deviation, N = 3

We hypothesized that the sequestration of siRNAs could be due to autophagy resulting from ER stress [36]. Autophagy was inhibited using the class III phosphoinositide 3-kinase inhibitor 3-methyladenine (3MA). The presence of 3MA in the media during transfection reduced the amount of siRNA that accumulated in cells to control levels, even with TM exposure (Fig. 2A). Autophagy inhibition reduced silencing in both TM-exposed and control cells, with the combination of TM and 3MA treatment reducing silencing most (Fig. 2B). 3MA impacts more than just autophagy, including other membrane formation events like endosome formation, which could explain the reduction in siRNA accumulation with 3MA treatment. Calculating the ***specific silencing*** or ratio of silencing achieved to the amount of siRNA accumulated in cells provides a measure of the likelihood of siRNAs to enter the cytoplasm and achieve silencing after entering the cell (micrographs of both stressed and unstressed cells show punctate signals indicating the majority of siRNA in both cases is sequestered in membrane-bound vesicles/compartments (Supplemental Fig. 2); thus, cellular accumulation alone is not sufficient to assess whether an siRNA can initiate silencing.). TM treatment altered specific silencing, while treatment with 3MA did not result in a significant change (Fig. 2C). This indicated that autophagy inhibition reduces the accumulation of siRNA while ER stress due to TM exposure alters intracellular processing of siRNA-containing complexes.

### Increased siRNA accumulation occurs with multiple initiators of ER Stress

**Fig. 3:**
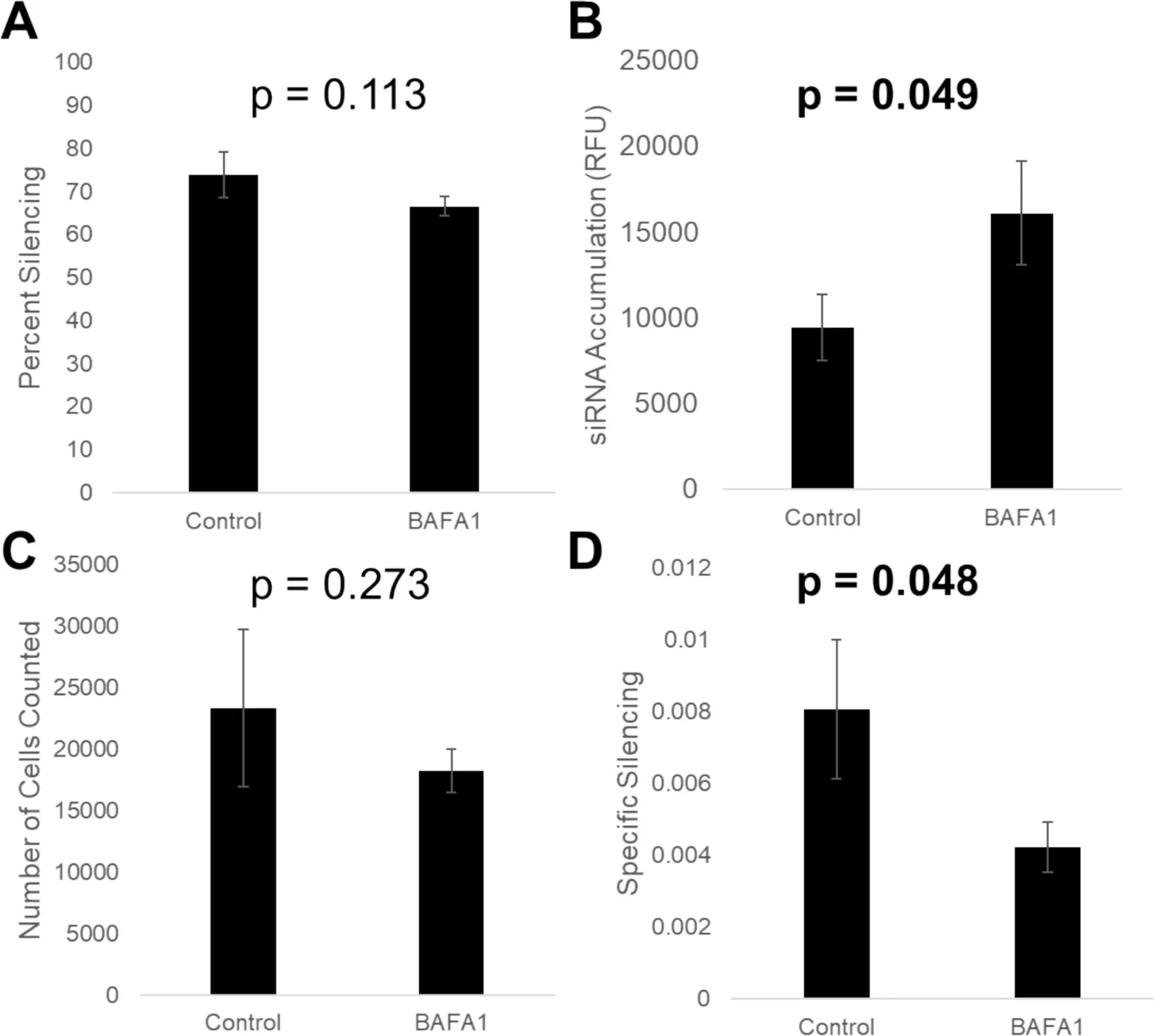
Bafilomycin A1 increases siRNA accumulation*.* **A**) siRNA accumulation in untreated and 50 nM BAFA1 treated cells. **B**) Silencing in control and 50 nM BAFA1 treated cells. **C**) Number of cells counted in control cells and 50 nM BAFA1 treated cells. **D**) Specific silencing in control cells and 50 nM BAFA1 treated cells. Values are pair-wise p-values calculated from Student’s T-tests, bold indicates *p* < 0.05. Error bars represent standard deviation, N = 3

Bafilomycin A1 (BAFA1) is a V-ATPase inhibitor that prevents the acidification of endosomes and prevents the maturation of early endosomes to late endosomes and lysosomes [37]. BAFA1 concentrations as low as 10 nM have been shown to induce ER stress, while concentrations above 1 µM disrupt glycosylation and activate CHOP by altering Golgi pH [38]. We therefore exposed cells to 50 nM BAFA1 (high enough to ensure ER stress activation while low enough to avoid CHOP activation) as an alternative ER stressor to determine if the effects on siRNA accumulation and silencing were comparable to the effects from TM exposure. As with TM, treatment with BAFA1 during transfection led to an increased intracellular accumulation of siRNA (Fig. 3A). Because silencing and cell number did not change significantly (Figs. 3B and C), specific silencing decreased (Fig. 3D). This shows that other ER stress inducers that indirectly impact glycosylation could have similar effects on siRNA accumulation to that seen in TM exposure. Additionally, based on the known role of BAFA1 in preventing maturation to late endosomes and lysosomes, the results also suggest that siRNAs are being sequestered in compartments upstream of late endosome/lysosome formation.

### ER stress prolongs siRNA accumulation and silencing

**Fig. 4:**
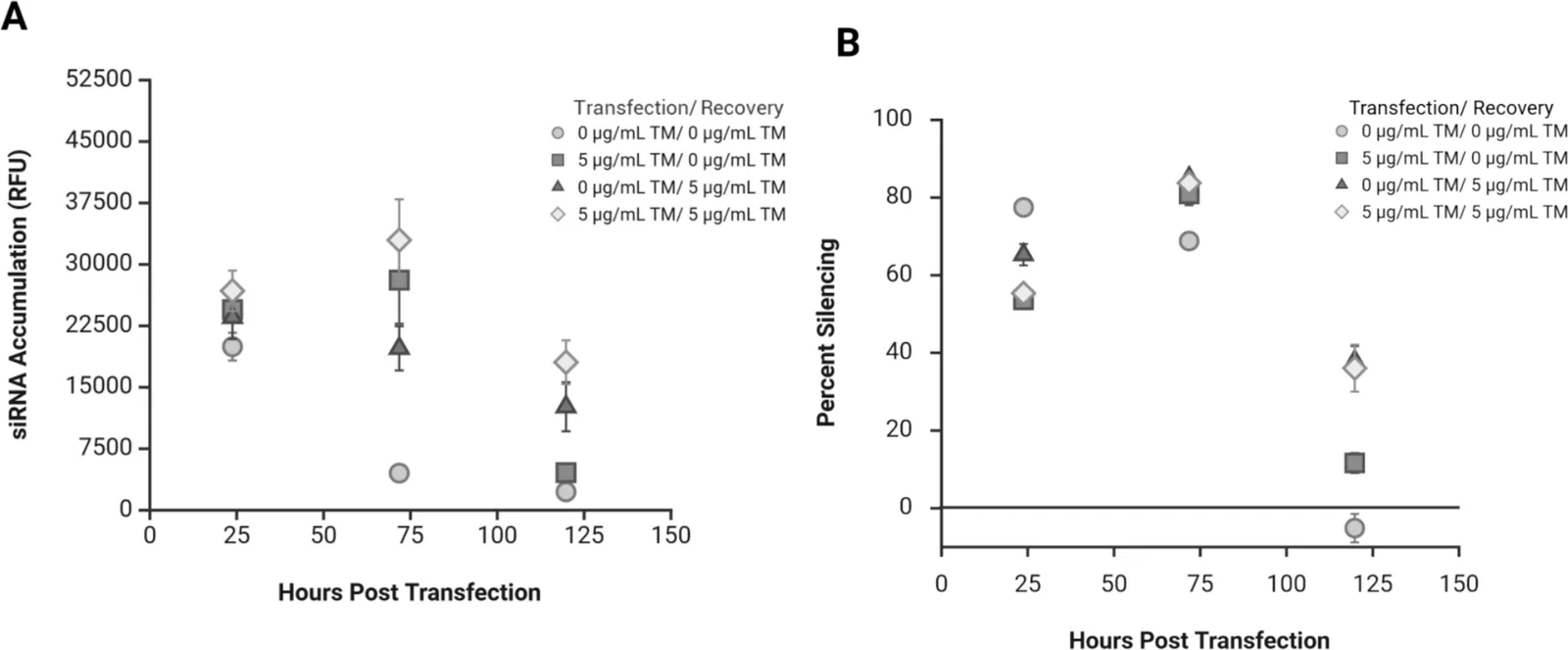
Duration of siRNA accumulation and EGFP silencing for unstressed (circles), TM during transfection (squares), TM during recovery (triangle), and TM during both transfection and recovery (diamonds). **A**) siRNA accumulation vs. time. **B**) EGFP percent silencing vs. time. Error bars are standard deviation, N = 3. Comparative p-values are in Supplemental Information Tables 1–4

Recognizing that siRNAs were being sequestered in ER-stressed cells, we investigated whether the siRNAs were degraded or remained intact and able to initiate silencing upon reaching the cytoplasm. Cells were transfected in media with or without TM and then allowed to recover in TM-containing culture media or TM-free culture media. 24 h post transfection, cells that received TM at any point had a higher accumulation of siRNA than control cells (Fig. 4A). At 72 h, siRNA fluorescence in control cells approached background, while the siRNA fluorescence remained elevated in cells treated at any time with TM (Fig. 4A, square, triangle, and diamond). Even cells that only received TM during transfection, the fluorescence remained elevated compared to control (Fig. 4A, square vs. circle), presumably due to the lingering consequences of ER stress signaling during the transfection period. At 120 h, cells that recovered in TM still retained more siRNA than control cells (Fig. 4A, triangle and diamond). Cells exposed to TM only during transfection finally returned to near-normal function, with siRNA accumulation only slightly greater than control cells (Fig. 4A, square vs. circle).

Simultaneous measurement of silencing showed that the siRNA accumulated in ER-stressed cells was still functional at later time points. In agreement with our prior experiments, at 24 h, control cells had the greatest silencing followed by cells that were exposed to TM during recovery (Fig. 4B, circle and triangle); cells that received TM during transfection had the lowest silencing (Fig. 4B, square and diamond). At 72 h, cells exposed to TM at any point (Fig. 4B, square, triangle, and diamond) had increased silencing that matched that seen in control cells at 24 h. At 120 h, as with siRNA accumulation, silencing was markedly lower for cells recovering in the absence of TM (Fig. 4B, circle and square), while cells exposed to TM during recovery maintained some degree of silencing (Fig. 4B, triangle and diamond). Taken together, these results indicate that sequestered siRNAs in the TM-treated cells are not being degraded and can eventually enter the cytoplasm to achieve silencing. Moreover, continued ER stress prolonged the sequestration of the siRNA-containing complexes without noticeably reducing their efficacy.

## Discussion

Here, we showed that ER stress and UPR induction altered the delivery and processing of siRNA-containing complexes in TM-treated HeLa cells. TM-induced ER stress increased the accumulation of siRNA while decreasing silencing. Our results also demonstrated that autophagy played a central role in the sequestration of delivered siRNAs. Further, we demonstrated that the sequestered siRNAs remained functional, with ER stress increasing the persistence of siRNA silencing.

Induction of the UPR is both stimulus- and cell line-dependent; however, there are many available ER stress models for UPR activation in cell culture [11]. TM initiates ER stress by inhibiting N-glycosylation. Many other models and physiological stressors also impact N-glycosylation [39]. In laboratory studies, BAFA1 and thapsagargin slowed glycosylation by increasing pH and decreasing ER calcium concentration respectively, thereby reducing the efficiency of glycosylation enzymes [40, 41]. Clinically, alterations in glycosylation are involved in a number of diseases. Rheumatoid diseases are associated with decreased activity of galactose transferase enzymes, which play a key role in N-glycosylation [42]. Metastatic cancers show increased sialylation and fucosylation, important glycosylation processes [43]. Neurological conditions can result from mutations in enzymes that reduce the production of phosphorylated sugars, resulting in unoccupied glycosylation sites and unstable proteins [44]. Thus, exposure to TM is a reasonable model for ER stress that occurs in some clinical situations where siRNA-based therapeutics might be applied.

Our results showed that TM-induced ER stress resulted in the accumulation of siRNA with a corresponding reduction in maximal silencing. While this result is counterintuitive, it adds to growing evidence that siRNA accumulation in a cell does not necessarily result in increased silencing [21, 45]. Lipid-based siRNA complexes, like those made from LF2K and LNPs, enter the cytoplasm via interactions of the delivery vehicle with the cell membrane [46] As such, we believe our results with LF2K and TM demonstrate that delivery of siRNAs with lipid-based nanoparticles will likely be impacted by ER stress. Other delivery strategies, such as poly(ethylenimine)-siRNA complexes, that utilize the “proton-sponge effect” for endosomal escape [23], may show stronger correlation between accumulation and silencing because, with these vehicles, interactions of the siRNA-containing complex with membranes is not required for entry into the cytoplasm [21]. We expect that the most clinically successful RNAi therapeutics, GalNAc-siRNA conjugates, are impacted by ER stress-induced autophagy, as they are internalized by receptor-mediated endocytosis, which relies on normal vesicle trafficking to recycle receptors to the cell membrane to achieve sufficient endocytosis of siRNA[47]. In contrast, changes in cell states that decrease membrane rigidity and polarity, like high unsaturated fatty acid content [28], may increase the ability of lipid-based siRNA complexes to merge with the endosomal membrane and improve cytoplasmic delivery.

Our results indicate that autophagy mediates siRNA sequestration but apparently does not damage the siRNA or prevent its function upon release to the cytoplasm. A previous study reported rapamycin, a mammalian Target of Rapamycin Complex 1 (mTORC1) inhibitor, induced autophagy and improved silencing in H1299 cells using LF2K while 3MA, which inhibits autophagy, reduced silencing [48]. Our results support that 3MA reduced silencing but further showed that there is a reduction in the accumulation of siRNA in the autophagy-inhibited cells. TM has been shown to activate and inhibit mTORC1 pathway in a context specific manner [49]. Because the specific silencing is consistent in the unstressed and stressed cells regardless of autophagy inhibition (Fig. 2C), it is possible that other UPR factors, TM-specific factors (e.g., changes in target gene expression, membrane rigidity, and glycoprotein function), or time-dependent changes (24 h vs. 48 h) are responsible for the discrepancy in silencing efficiency reported in this study and that reported using rapamycin [48].

Our results showed that BAFA1 increased siRNA accumulation without impacting silencing. Low concentrations of BAFA1 prevent acidification without causing ER stress, while high concentrations of BAFA1 led to GRP-78, CHOP, and mTOR activation [38]. The fact that high concentration BAFA1 impacts siRNA delivery similarly to TM in our cells shows that the impact of ER stress on siRNA accumulation is not specific to TM exposure. siRNA release to the cytoplasm occurs during endosome maturation [46], but we showed that ER stress slows or blocks this maturation in favor of autophagy. Thus, we believe that TM causes a disruption in maturation from the early endosomes to late endosomes/lysosomes and promotes trafficking to a more autophagosome-like vesicle.

At 24 h, ER stress and TM reduced silencing despite higher siRNA accumulation. In contrast, at 72 and 120 h, autophagy and ER stress increased siRNA accumulation and silencing. While it is possible that siRNAs were degraded while the Cy5 tag remained providing a false positive measure of siRNA levels, the rescue of silencing at 72 h and increased silencing at 120 h indicates that, for cells recovering in TM-free media after TM-exposed transfection, at least some siRNA continued to be functional (Fig. 4). Thus, ER stress can influence the processing characteristics of siRNA-containing complexes, without fully eliminating siRNA function. Comorbidities that increase ER stress, especially those that activate the mTOR pathway, will likely delay silencing despite siRNA accumulation in the tissues. While this could be advantageous in achieving more persistent silencing, it could result in increased toxicity due to excessive siRNA accumulation. Each of these factors must be accounted for in designing clinical siRNA delivery strategies and dosing schedules.

### Limitations

In this work, we delivered Cy5-tagged siRNA targeting constitutively expressed EGFP to HeLa cells using Lipofectamine 2000 and used TM as an ER stress inducer. We recognize that endocytosis and processing of lipoplexes differs from delivery by other mechanisms, such as GalNAc-conjugated siRNAs or polymeric delivery [47, 50]. Nonetheless, given our comprehensive understanding of how lipoplex delivery depends on endocytosis [45], we believe that delivery using Lipofectamine could be markedly altered by disruptions to the endomembrane system due to ER stress. We acknowledge the possibility that our results may not be generalizable to all approaches for siRNA delivery. Other ER stress inducers, particularly non-autophagy inducing and mitochondrial-stress associated stressors, may also have distinct impacts on siRNA processing. The experiments contained are done in cell culture to model individual cell interactions with siRNA complexes; therefore, limitations from in vivo delivery were beyond the scope of this work. That said, we expect that in vivo delivery would result in increased and more persistent accumulation of siRNAs in the liver, a primary organ responsible for siRNA clearance [9] with cells often experiencing ER stress [51], which would in turn result in more persistent effects. Acknowledging these limitations, our results nonetheless demonstrate a need to consider the impact of disease state on siRNA delivery and dosing in therapeutic and laboratory applications.

## Supplementary Information

Below is the link to the electronic supplementary material.

ESM 1 (DOCX 225 KB)

## Data Availability

The data that support the findings of this study are available from the corresponding author upon reasonable request.

## Declarations

### Data sharing

The data that support the findings of this study are available from the corresponding author upon reasonable request.
