# Supplementary material for: Endoplasmic reticulum stress induced autophagy alters cellular processing of cationic lipid delivered siRNAs
Source: Drug Deliv Transl Res. 2026 Mar 17;16(11):4198–207. doi: 10.1007/s13346-026-02096-8 (PMC13748136; doi:10.1007/s13346-026-02096-8)
Supplement: Supplementary file 1 [file 13346_2026_2096_MOESM1_ESM.docx]

**Supplemental Figure 1** EGFP expression measured in raw fluorescence units (RFU) by flow cytometry in HeLa cells constitutively expressing EGFP with increasing concentrations of tunicamycin in control cells (light grey circles) and cells exposed to siRNA (dark grey squares). All untreated cells express more EGFP than the treated cells at 0 µg/mL tunicamycin cells. Thus, the reduction in EGFP level due to siRNA silencing exceeds that due to exposure to tunicamycin alone.

**Supplemental Figure 2** Micrographs of HeLa-EGFP cells transfected with Dy547-tagged siRNA-Lipofectamine 2000 complexes taken on a Nikon Eclipse Ts2R-FL Inverted Microscope. 24 hours after cells were transfected in A) 0 µg/mL tunicamycin or B) 5 µg/mL tunicamycin, cells were washed twice with DBPS before being live-cell imaged in DBPS. Gray represents the EGFP mask of the cells and red represents the Dy547 signal. In both cases, siRNA signal is punctate within cells indicating the siRNA is localized to membrane-enclosed compartments.

**Supplemental Table 1:** siRNA accumulation p-values, comparisons by TM exposure during transfection/TM exposure during recovery at each time point

| 24 hrs | 5 µg/mL TM/ 0 µg/mL TM | 0 µg/mL TM/ 5 µg/mL TM | 5 µg/mL TM/ 5 µg/mL TM |
| --- | --- | --- | --- |
| 0 µg/mL TM/ 0 µg/mL TM | 0.023072409 | 0.143992618 | 0.029313301 |
| 5 µg/mL TM/ 0 µg/mL TM |  | 0.589309205 | 0.220227406 |
| 0 µg/mL TM/ 5 µg/mL TM |  |  | 0.219844613 |
| 72 hrs | 5 µg/mL TM/ 0 µg/mL TM | 0 µg/mL TM/ 5 µg/mL TM | 5 µg/mL TM/ 5 µg/mL TM |
| 0 µg/mL TM/ 0 µg/mL TM | 0.004653454 | 0.00241054 | 0.00223491 |
| 5 µg/mL TM/ 0 µg/mL TM |  | 0.094224384 | 0.329273153 |
| 0 µg/mL TM/ 5 µg/mL TM |  |  | 0.027311285 |
| 120 hrs | 5 µg/mL TM/ 0 µg/mL TM | 0 µg/mL TM/ 5 µg/mL TM | 5 µg/mL TM/ 5 µg/mL TM |
| 0 µg/mL TM/ 0 µg/mL TM | 0.006631362 | 0.009402819 | 0.002016419 |
| 5 µg/mL TM/ 0 µg/mL TM |  | 0.019505464 | 0.003321534 |
| 0 µg/mL TM/ 5 µg/mL TM |  |  | 0.100401916 |

**Supplemental Table 2:** siRNA accumulation p-values, comparisons by time point for each stress case - TM exposure during transfection/TM exposure during recovery

| 0 µg/mL TM/ 0 µg/mL TM | 72 hrs | 120 hrs |
| --- | --- | --- |
| 24 hrs | 0.000636858 | 0.000385536 |
| 72 hrs |  | 0.008180459 |
| 5 µg/mL TM/ 0 µg/mL TM | 72 hrs | 120 hrs |
| 24 hrs | 0.328039668 | 3.1722E-05 |
| 72 hrs |  | 0.004681044 |
| 0 µg/mL TM/ 5 µg/mL TM | 72 hrs | 120 hrs |
| 24 hrs | 0.182844105 | 0.017948896 |
| 72 hrs |  | 0.054653534 |
| 5 µg/mL TM/ 5 µg/mL TM | 72 hrs | 120 hrs |
| 24 hrs | 0.149007243 | 0.025568286 |
| 72 hrs |  | 0.019659141 |

**Supplemental Table 3:** silencing p-values, comparisons by TM exposure during transfection/TM exposure during recovery at each time point

| 24 hrs | 5 µg/mL TM/ 0 µg/mL TM | 0 µg/mL TM/ 5 µg/mL TM | 5 µg/mL TM/ 5 µg/mL TM |
| --- | --- | --- | --- |
| 0 µg/mL TM/ 0 µg/mL TM | 2.34309E-05 | 0.004931266 | 3.19591E-05 |
| 5 µg/mL TM/ 0 µg/mL TM |  | 0.005619926 | 0.048594949 |
| 0 µg/mL TM/ 5 µg/mL TM |  |  | 0.008877474 |
| 72 hrs | 5 µg/mL TM/ 0 µg/mL TM | 0 µg/mL TM/ 5 µg/mL TM | 5 µg/mL TM/ 5 µg/mL TM |
| 0 µg/mL TM/ 0 µg/mL TM | 0.007162321 | 0.000270762 | 0.00031916 |
| 5 µg/mL TM/ 0 µg/mL TM |  | 0.097723923 | 0.202762249 |
| 0 µg/mL TM/ 5 µg/mL TM |  |  | 0.053474072 |
| 120 hrs | 5 µg/mL TM/ 0 µg/mL TM | 0 µg/mL TM/ 5 µg/mL TM | 5 µg/mL TM/ 5 µg/mL TM |
| 0 µg/mL TM/ 0 µg/mL TM | 0.00749265 | 0.000803959 | 0.002080227 |
| 5 µg/mL TM/ 0 µg/mL TM |  | 0.002299988 | 0.007615018 |
| 0 µg/mL TM/ 5 µg/mL TM |  |  | 0.690049421 |

**Supplemental Table 4:** silencing p-values, comparisons by time point for each stress case - TM exposure during transfection/TM exposure during recovery

| 0 µg/mL TM/ 0 µg/mL TM | 72 hrs | 120 hrs |
| --- | --- | --- |
| 24 hrs | 0.001892849 | 3.92131E-05 |
| 72 hrs |  | 6.18457E-05 |
| 5 µg/mL TM/ 0 µg/mL TM | 72 hrs | 120 hrs |
| 24 hrs | 0.000554978 | 0.000106 |
| 72 hrs |  | 7.55522E-05 |
| 0 µg/mL TM/ 5 µg/mL TM | 72 hrs | 120 hrs |
| 24 hrs | 0.001174129 | 0.002148554 |
| 72 hrs |  | 0.000243805 |
| 5 µg/mL TM/ 5 µg/mL TM | 72 hrs | 120 hrs |
| 24 hrs | 9.84767E-06 | 0.01186891 |
| 72 hrs |  | 0.000856419 |
